# Short-term heart rate variability is preserved in Parkinson’s disease under atomoxetine

**DOI:** 10.1093/braincomms/fcag338

**Published:** 2026-09-08

**Authors:** Isabella F Orlando, Frank H Hezemans, Kamen A Tsvetanov, Rong Ye, Catarina Rua, Ralf Regenthal, Roger A Barker, Caroline H Williams-Gray, Luca Passamonti, Trevor W Robbins, James B Rowe, Claire O’Callaghan

**Affiliations:** School of Medical Sciences, Faculty of Medicine and Health, University of Sydney, Sydney 2050, New South Wales, Australia; MRC Cognition and Brain Sciences Unit, University of Cambridge, Cambridge CB2 7EF, UK; Department of Clinical Neurosciences and Cambridge University Hospitals NHS Trust, University of Cambridge, Cambridge CB2 0SZ, UK; Department of Clinical Neurosciences and Cambridge University Hospitals NHS Trust, University of Cambridge, Cambridge CB2 0SZ, UK; Department of Psychology, University of Cambridge, Cambridge CB2 3EA, UK; Department of Clinical Neurosciences and Cambridge University Hospitals NHS Trust, University of Cambridge, Cambridge CB2 0SZ, UK; Department of Clinical Neurosciences and Cambridge University Hospitals NHS Trust, University of Cambridge, Cambridge CB2 0SZ, UK; Division of Clinical Pharmacology, Rudolf-Boehm-Institute for Pharmacology and Toxicology, University of Leipzig, Leipzig 69978, Germany; Department of Clinical Neurosciences and Cambridge University Hospitals NHS Trust, University of Cambridge, Cambridge CB2 0SZ, UK; John van Geest Centre for Brain Repair, Department of Clinical Neurosciences, University of Cambridge, Cambridge CB2 0SZ, UK; John van Geest Centre for Brain Repair, Department of Clinical Neurosciences, University of Cambridge, Cambridge CB2 0SZ, UK; Department of Clinical Neurosciences and Cambridge University Hospitals NHS Trust, University of Cambridge, Cambridge CB2 0SZ, UK; Department of Psychology, University of Cambridge, Cambridge CB2 3EA, UK; Behavioural and Clinical Neuroscience Institute, University of Cambridge, Cambridge CB2 3EA, UK; MRC Cognition and Brain Sciences Unit, University of Cambridge, Cambridge CB2 7EF, UK; Department of Clinical Neurosciences and Cambridge University Hospitals NHS Trust, University of Cambridge, Cambridge CB2 0SZ, UK; School of Medical Sciences, Faculty of Medicine and Health, University of Sydney, Sydney 2050, New South Wales, Australia

**Keywords:** Parkinson’s disease, noradrenaline, atomoxetine, heart rate variability, locus coeruleus

## Abstract

Repurposed noradrenergic drugs have been proposed to treat neuropsychiatric symptoms in Parkinson’s disease and related conditions. While there is evidence that these drugs can be beneficial for cognition in selected patients, questions remain about their cardiovascular effects. Here, we tested whether heart rate variability (HRV) is altered in people with Parkinson’s disease, following a single-dose challenge with the noradrenaline reuptake inhibitor atomoxetine (40 mg, oral). Consistent with previous work, our cohort of people with idiopathic Parkinson’s disease (*n* = 15) had lower HRV than healthy controls (*n* = 22). Select markers of decreased HRV in people with Parkinson’s disease were associated with reduced integrity of the caudal locus coeruleus, measured using neuromelanin-sensitive ultra-high-field 7T magnetic resonance imaging. Following a randomized double-blind placebo-controlled crossover challenge in the Parkinson’s disease group, short-term resting HRV was not significantly altered following atomoxetine. Using Bayesian statistical inference, we demonstrated confidence in the preservation of HRV across measures in the time, frequency and non-linear domains. Our findings are in favour of a safe cardiovascular profile for atomoxetine in Parkinson’s disease, further supporting noradrenergic modulation as a viable treatment strategy for neuropsychiatric symptoms in Parkinson’s disease and related disorders.

## Teaser

There is a renewed interest in using noradrenergic drugs to treat cognitive and psychiatric symptoms in Parkinson’s disease and related disorders. The authors show that heart rate variability is preserved in people with Parkinson’s disease receiving an acute dose of atomoxetine, supporting a safe cardiovascular profile for the drug.

## Graphical Abstract

**Graphical Abstract:**
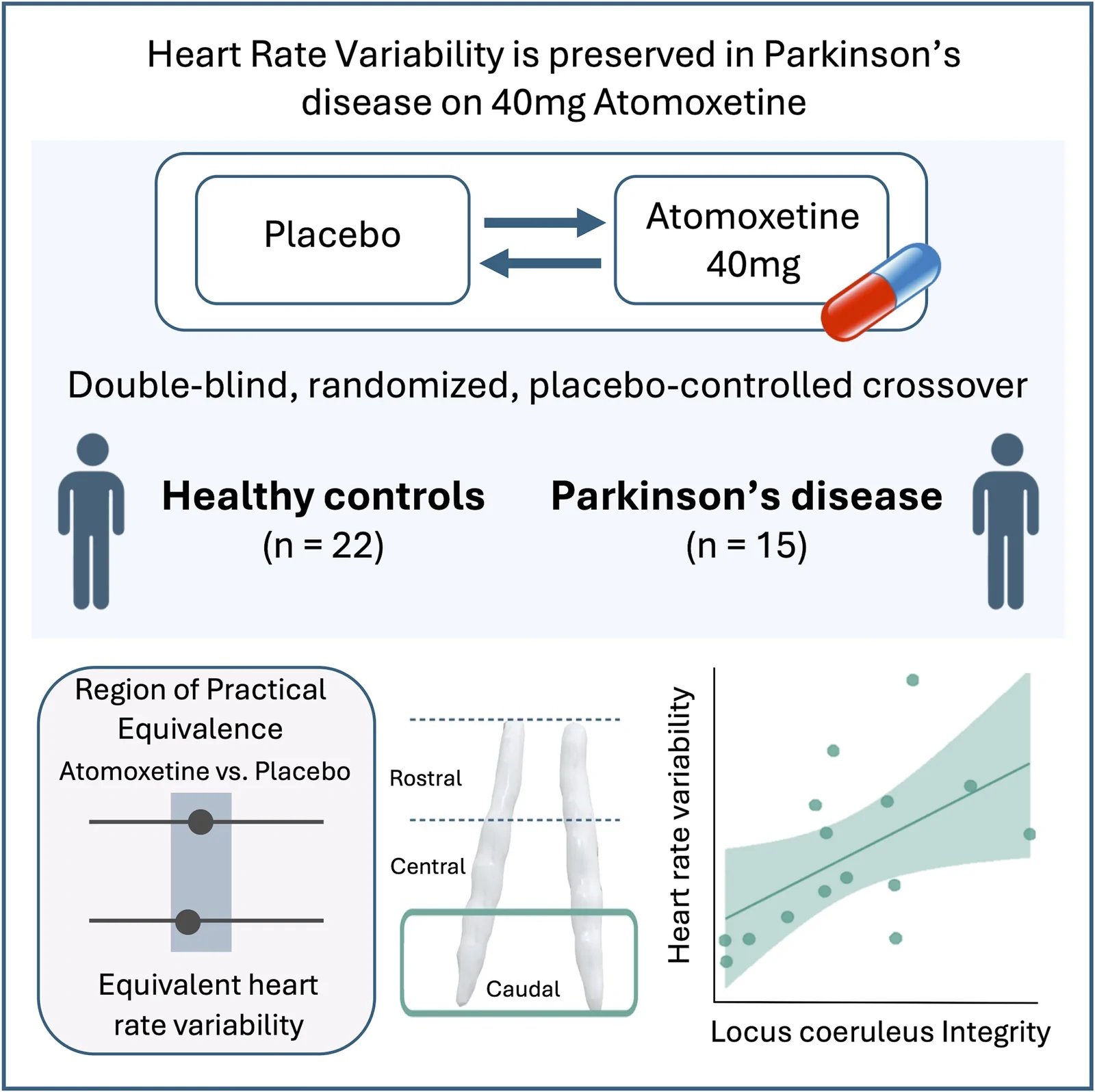

## Introduction

The noradrenergic locus coeruleus is one of the earliest sites of pathology in Parkinson’s disease and is implicated in both motor and non-motor symptoms.^1-3^ Repurposed noradrenergic drugs have been reported to improve cognitive and psychiatric symptoms in some individuals,^4-8^ with the possibility that they may also have disease-modifying effects.^9,10^ However, autonomic problems in Parkinson’s disease raise critical questions about the safety and cardiovascular effects of noradrenergic treatment approaches.

Of particular interest is the noradrenaline reuptake inhibitor atomoxetine, which increases extracellular levels of noradrenaline^11^ and phasic-to-tonic locus coeruleus firing.^12^ Atomoxetine is licensed in many countries for the treatment of attention-deficit hyperactivity disorder in children and adults. The cardiovascular safety of atomoxetine is well described in these patient groups. The drug is generally very well tolerated and associated with only small increments in blood pressure or pulse rate^13^ that are not linked with serious adverse cardiac events.^14,15^ Atomoxetine is tolerated well in Parkinson’s disease following acute challenges^5,7,8^ and longer-term trials.^16-18^ Reported adverse events were rare, but included cardiovascular side-effects (i.e. altered blood pressure or pulse rate) and gastrointestinal side-effects of atomoxetine (i.e. nausea/vomiting, dry mouth, decreased appetite and constipation). Severe arrhythmias are extremely rare but remain a theoretical concern, particularly for use in older adults. However, the possibility of more nuanced cardiovascular changes under atomoxetine in Parkinson’s disease has not been tested. This is particularly relevant, given the cardiac and autonomic symptoms associated with Parkinson’s disease.

Noradrenergic sympathetic innervation of the heart is affected in Parkinson’s disease, resulting in profound myocardial noradrenaline deficiency.^19^ Cardiac sympathetic denervation (e.g. measured by ^123^I-metaiodobenzylguanidine scintigraphy) supports the diagnosis of Parkinson’s disease^20^ and progresses with disease severity.^21^ Nuclei that provide central control of cardiac function are affected in Parkinson’s disease, including the locus coeruleus, which plays a major role in cardiovascular regulation.^22^ Cardiovascular autonomic dysfunction in Parkinson’s disease can manifest as abnormal blood pressure (e.g. labile blood pressure or orthostatic hypotension)^23^ or changes in the dynamic, adaptive regulation of heart rhythms. The latter is captured by measures of heart rate variability (HRV), which is reduced in Parkinson’s disease.^24^ While previous longer-term trials^16-18^ and acute challenges^5,8^ of atomoxetine in Parkinson’s disease established cardiovascular tolerability using primary measures of blood pressure and pulse rate, no study to date has explored the drug’s effect on HRV, which offers a more sensitive and comprehensive measure of autonomic state. In contrast to previous studies using frequentist approaches, the Bayesian framework we employ further allows us to quantify evidence in favour of cardiovascular stability, rather than merely failing to detect an effect.

Here, we measured short-term resting HRV in people with Parkinson’s disease undergoing an acute, single-dose challenge with 40 mg of oral atomoxetine or placebo. Given the tolerability of atomoxetine in Parkinson’s disease and its mild cardiovascular profile in attention-deficit hyperactivity disorder, we predicted that we would not see significant HRV changes under the drug. Bayesian statistical inference was used, given the predicted null effect and given its advantages in a small sample size, including the incorporation of priors and generation of full posterior distributions that allow for a more direct quantification of uncertainty. Analyses first focussed on the atomoxetine versus placebo comparison (i.e. the drug effect) and then on comparisons between Parkinson’s disease (on placebo) versus healthy controls (i.e. interpreted as a disease effect). Participants underwent neuromelanin-sensitive neuroimaging with 7T MRI, providing a marker of locus coeruleus integrity. We compared locus coeruleus integrity against the HRV metrics that were altered in people with Parkinson’s compared to controls, to determine whether disease-related changes in HRV relate to reduced locus coeruleus integrity.

## Materials and methods

General study design and participant characteristics overlap with previous publications.^4,6,7,25^ The study was approved by the local Ethics Committee (REC 10/H0308/34), and participants provided written informed consent according to the Declaration of Helsinki. The study is registered on the ISRCTN registry with study ID ISRCTN46299660 (https://doi.org/10.1186/ISRCTN46299660).

### Participants

Nineteen individuals with Parkinson’s disease were recruited through the University of Cambridge Parkinson’s disease research clinic and Parkinson’s UK volunteer panels. Participants met United Kingdom Parkinson’s Disease Society Brain Bank criteria, were aged between 50 and 80 years, had Hoehn and Yahr stages of 1.5–3 and had no dementia and contraindications to atomoxetine or 7T MRI. Twenty-six age-, sex- and education-matched healthy control participants were recruited from local volunteer panels. Control participants were screened for a history of psychiatric or neurological disorders and were not taking psychoactive or noradrenergic medications. Following ECG data preprocessing and signal-quality checks (described below), the final sample analysed included 15 people with Parkinson’s disease and 22 controls.

### Study procedure

Participants with Parkinson’s disease were tested over three sessions. Session 1 included MRI scanning and baseline clinical assessment of cognition and motor function. Sessions 2 and 3 were a double-blind, placebo-controlled crossover design, with participants randomized to receive 40 mg of atomoxetine or placebo. Drug/placebo order was randomly permuted in groups of six successive recruits. We randomized three participants to placebo-first and three to atomoxetine-first, within each successive block of 1–6, 7–12, 13–18 and 19–24. This approach guards against unequal randomization that can arise through randomizing across the entire group. The visits were scheduled ≥6 days apart (mean = 7.4 days; SD 1.8 days; range 6–14 days). At each visit, blood samples were taken 2 h after drug/placebo administration, corresponding to the predicted peak in plasma concentration following an oral atomoxetine dose.^26^ Mean plasma concentration^27^ was 264.07 ng/ml after atomoxetine (SD = 124.50 ng/ml, range: 90.92–595.11 ng/ml) and 0 ng/ml after placebo. Participants then commenced a battery of computerized cognitive tasks that took approximately 2 h; ECG recording was performed during a rest break midway through the testing session. Supine/lying and sitting upright blood pressure and pulse rate measures were monitored three times during the sessions (on arrival, 2 h after tablet administration, and on completion of testing). Subjective measures of mood were collected prior to tablet administration and 2 h post-administration, using a set of 16 visual analogue scales.^28^ All sessions were completed at a similar time of day, with participants on their regular anti-parkinsonian medications. Control participants provided normative data and did not undergo the atomoxetine/placebo manipulation. They were tested in a single session that included MRI scanning and the same experimental task battery undertaken by the Parkinson’s disease group.

### ECG recording

Ten-minute ECG recordings were collected with participants sitting upright in a chair. Short-term HRV refers to metrics derived from recordings of approximately 5–10 min, in contrast to long-term assessment over ∼24 h.^29,30^ Our 10-min window falls within the short-term category and provides sufficient resolution for spectral estimation down to the very low-frequency band. ECG was recorded using a BIOPAC MP36 system (BIOPAC Systems, Inc., USA) at a sampling frequency of 1000 Hz. Recordings were obtained using disposable electrodes attached to one forearm and both ankles. Participants were asked to sit still and refrain from talking.

### ECG data preprocessing and extraction of HRV metrics

ECG data preprocessing and extraction of HRV metrics used the PhysioNet Cardiovascular Signal Toolbox,^31^ implemented in MATLAB (R2019b). The toolbox prepares ECG recordings for HRV estimation via detection and removal of data based on arrhythmias (including automatized detection of atrial fibrillation and premature ventricular contraction), noise and low signal quality. Data were analysed in a single 10-min window. Using the default parameter settings as a guide,^31^ we set less conservative thresholds to account for possible signal degradation in an older, neurodegenerative disease cohort (see Supplementary material for parameter settings). This approach is consistent with the toolbox recommendations to adjust parameter settings to accommodate the population under investigation; the thresholds we set are consistent with prior application of the toolbox in a Parkinson’s disease cohort.^32^ Based on these criteria, four healthy controls and four participants with Parkinson’s disease (for both their placebo and atomoxetine sessions) were excluded. HRV metrics were then calculated on the normal-to-normal (NN) intervals, including time domain, frequency domain and non-linear metrics. Time-domain metrics quantify the amount of variability in the inter-beat intervals; frequency-domain metrics estimate absolute or relative power within frequency bands; non-linear metrics quantify the unpredictability, or complexity, of the time series.^30^ See Table 1 and Supplementary material for descriptions of each metric.

**Table 1:** HRV metrics

Domain	Measure	Description
Time	SDNN	Standard deviation of normal-to-normal intervals (ms)
	RMSSD	Root mean square of successive differences of R–R intervals (ms)
	AC	Acceleration capacity (ms)
	DC	Deceleration capacity (ms)
Frequency	vLF	Power in very-low-frequency range (0.003 < vLF < 0.04 Hz) (ms^2^)
	LF	Power in low-frequency range (0.04 Hz <LF < 0.15 Hz) (ms^2^)
	HF	Power in high-frequency range (0.15 < HF < 0.4 Hz) (ms^2^)
	LF:HF	LF (ms^2^)-to-HF (ms^2^) ratio
Non-linear	SD1	Standard deviation of the distance of each point from the y = −x axis on the Poincaré plot (ellipse width)
	SD2	Standard deviation of the distance of each point from the y = x axis on the Poincaré plot (ellipse length)
	SD1:SD2	SD1/SD2 ratio (ms)
	Sample entropy	Measure of the regularity and complexity of a timeseries (a.u.), with higher values indicating less regularity and more complexity

### Statistical analysis

We performed Bayesian estimation of linear mixed-effects models to evaluate group differences in HRV metrics. This approach offers several advantages over frequentist methods that are particularly relevant, given our study design and relatively small sample sizes. Specifically, Bayesian inference provides a principled framework for quantifying uncertainty and allows for the incorporation of domain expertise or regularization via prior distributions. In our case, priors were specified to be ‘weakly informative’, such that they provide moderate regularization and help stabilize computation, without imposing strong prior beliefs.

For each HRV metric, we performed two separate analyses. First, to assess drug effects, we compared people with Parkinson’s disease on atomoxetine versus placebo. Second, to assess disease-related effects, we compared people with Parkinson’s on placebo versus controls (noting that the control and patient groups not only differed by disease, but also in repeated-measures testing, and the expectation of a possible drug treatment effect). Each model included a fixed effect of condition, operationalized as drug status (atomoxetine versus placebo) or group (Parkinson’s disease versus control), depending on the contrast of interest. A random intercept for participants was included to account for repeated measures within individuals. In models testing drug effects, we included a fixed effect of visit order (placebo-first versus atomoxetine-first) as a covariate of no interest.

Following recommendations for regularizing inference in small samples,^33^ we used Student’s *t* distributions with 3 degrees of freedom as priors on all fixed-effect regression coefficients and on the residual standard deviation. For each model, the parameters of the Student’s *t* priors (location and scale) were adjusted based on the empirical distribution of the outcome variable in the reference group of the comparison. This approach was informed by the default method of prior elicitation used in the *rstanarm* package.^34^ Thus, for models analysing a drug effect, the priors were based on the patient group on placebo; for models analysing a disease-related effect, the priors were based on the control group. For all regression coefficients and for the residual SD, the scale parameter of the prior was set to the empirical SD of the reference group. For the intercept, the prior’s location parameter was set to the empirical mean of the reference group; for all other coefficients, the prior’s location parameter was set to zero.

Model fitting was implemented in R (version 4.2.2) using the *brms* package,^35^ which provides an interface to *Stan*^36^ for Hamiltonian Monte Carlo sampling. We used four chains of 5000 iterations each, discarding the first 1000 iterations as warm-up. Convergence diagnostics, including the potential scale reduction factor ($\hat{R}$), were inspected to ensure validity of the posterior samples. For each regression coefficient, we report the mean of its posterior distribution as the estimated effect size, and the 95% quantile interval as the credible interval (CI), representing a range of plausible effect sizes.

We used the Region of Practical Equivalence (ROPE) to assess evidence of group differences. The ROPE defines a range of values considered negligible—that is, practically equivalent to a null effect.^37^ Here, the ROPE was defined as ±0.1 times the empirical SD of the control group.^38^ If the posterior mean lies outside the ROPE, there is evidence to accept the hypothesis of a difference between groups; if the CI is outside of the ROPE, the evidence for accepting a difference is strong.^38,39^ If the posterior mean is inside the ROPE, this is evidence in favour of the null (i.e. no difference between groups); if the CI lies entirely within the ROPE, then the null hypothesis of no group difference can be accepted.^38,39^

To compare demographic and clinical measures, in order to confirm patient and control groups were reasonably matched, we conducted Bayesian independent-samples *t*-tests and contingency table analysis using the *BayesFactor* package.^40^ We report the Bayes factor (BF) for the alternative hypothesis (i.e. non-zero group difference) over the null hypothesis, with BF >1 indicating relative evidence for the alternative hypothesis and BF >3 indicating positive evidence for the alternative hypothesis.^41^

### Locus coeruleus integrity estimation and relationship with HRV

As part of this study, participants underwent MRI scanning on a 7 T Magnetom Terra (Siemens, Erlangen, Germany) with a 32-channel head coil (Nova Medical, Wilmington, MA, USA). The locus coeruleus was imaged using a neuromelanin-sensitive 3D magnetization transfer–weighted sequence^42^ at high resolution (0.4 × 0.4 × 0.5 mm^3^). Locus coeruleus integrity was estimated as a contrast-to-noise ratio (CNR), derived using pipelines described in O’Callaghan *et al*.^7^ and Ye *et al*.,^43^ and reported in Supplementary material.

Previous reports from the current study cohort identified significantly reduced integrity, specifically in the caudal locus coeruleus, in people with Parkinson’s disease versus controls,^7,44^ which is replicated in other studies using neuromelanin-sensitive MRI.^45,46^ Since the caudal locus coeruleus is implicated in descending vagal projections,^22^ we tested whether caudal locus coeruleus integrity in people with Parkinson’s disease related to HRV measures—focusing on those HRV measures that differed convincingly between Parkinson’s participants and controls. We assessed the correlation using Bayesian tests of linear correlation implemented using the *BayesFactor* package^40,47^; these analyses were exploratory, and no correction for multiple comparisons was applied.

## Results

### Participant characteristics

Parkinson’s disease and control groups were matched for age; years of education; and scores on the Revised Addenbrooke’s Cognitive Examination, Mini-Mental State Examination and Montreal Cognitive Assessment (all BFs < 1; see Table 2). Within the drug and placebo sessions, there was no convincing evidence of a change in pulse rate. There was evidence of slightly elevated blood pressure on atomoxetine for the supine diastolic measure, specifically: placebo [mean (range); SD]: arrival = 70.40 (50–84; 9.50); 2 h = 67.60 (58–80; 5.08); completion = 72.67 (59–87; 8.00); atomoxetine: arrival = 74.07 (55–85; 7.42); 2 h = 74.33 (58–94; 8.50); and completion = 78.00 (63–98; 10.43). This observed difference on atomoxetine was not considered clinically significant and there was no evidence of elevated blood pressure across lying systolic or sitting measures. There was no change in subjective ratings of mood and arousal levels within the sessions. All pulse rate, blood pressure and subjective rating analyses are described in detail in Supplementary material (see Supplementary Table 1 and Supplementary Figs 1 and 2).

**Table 2:** Demographics and clinical assessments of participants in their regular medication state

	PD (*n* = 15)	Control (*n* = 22)	BF
Age (years)	67.20 (7.66)	65.41 (5.71)	0.418
Education (years)	14.27 (1.98)	14.96 (3.19)	0.400
Male/female	12/3	12/10	2.302
MMSE	29.47 (0.75)	29.73 (0.55)	0.578
MoCA	28.20 (1.78)	28.68 (1.39)	0.449
ACE-R total	95.53 (3.83)	97.55 (3.35)	0.967
MDS-UPDRS III	27.40 (12.77)		
Hoehn and Yahr stage	2.33 (0.49)		
Disease duration (years)	3.81 (1.55)		
Levodopa equivalent daily dose (mg/day)	521.27 (444.99)		

Data are presented as mean (SD). Comparisons of patient and control groups were performed with Bayesian *t*-tests. BF >3 indicates substantial evidence of a group difference. Levodopa equivalent daily dose was calculated using Tomlinson *et al*.^48^

MMSE, Mini-Mental State Examination; MoCA, Montreal Cognitive Assessment; ACE-R Total, Revised Addenbrooke’s Cognitive Examination Total score; MDS-UPDRS III, Movement Disorders Society Unified Parkinson’s Disease Rating Scale Part III.

#### Evidence for preserved HRV in Parkinson’s disease on atomoxetine versus placebo

**Figure 1:**
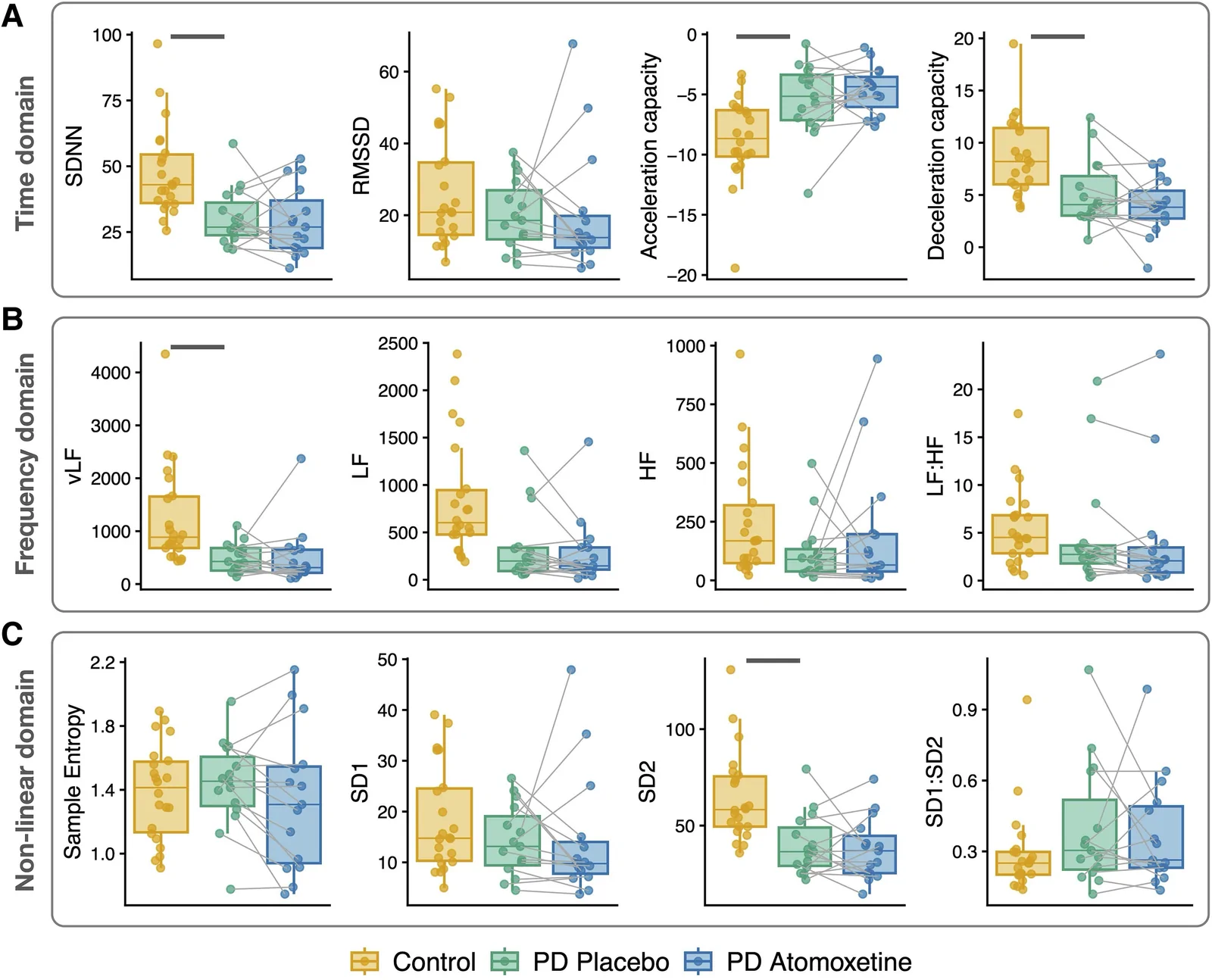
**Subject-level values for HRV metrics.** Boxplots show the median (horizontal line) and interquartile range, with data points representing individual participants (*N* = 22 Controls; *N* = 15 people with Parkinson’s disease who underwent both placebo and atomoxetine sessions), for (**A**) Time domain, (**B**) Frequency domain and (**C**) Non-linear domain. Grey bars indicate metrics for which the Bayesian regression models reported in Figs 2 and 3 provided evidence of a group difference (i.e. the posterior mean fell outside the ROPE and the 95% CI excluded zero). SD1 = standard deviation of ellipse width on the Poincaré plot; SD2 = standard deviation of ellipse length on the Poincaré plot; SD1:SD2 = SD1-to-SD2 ratio; PD = Parkinson’s disease.

**Figure 2:**
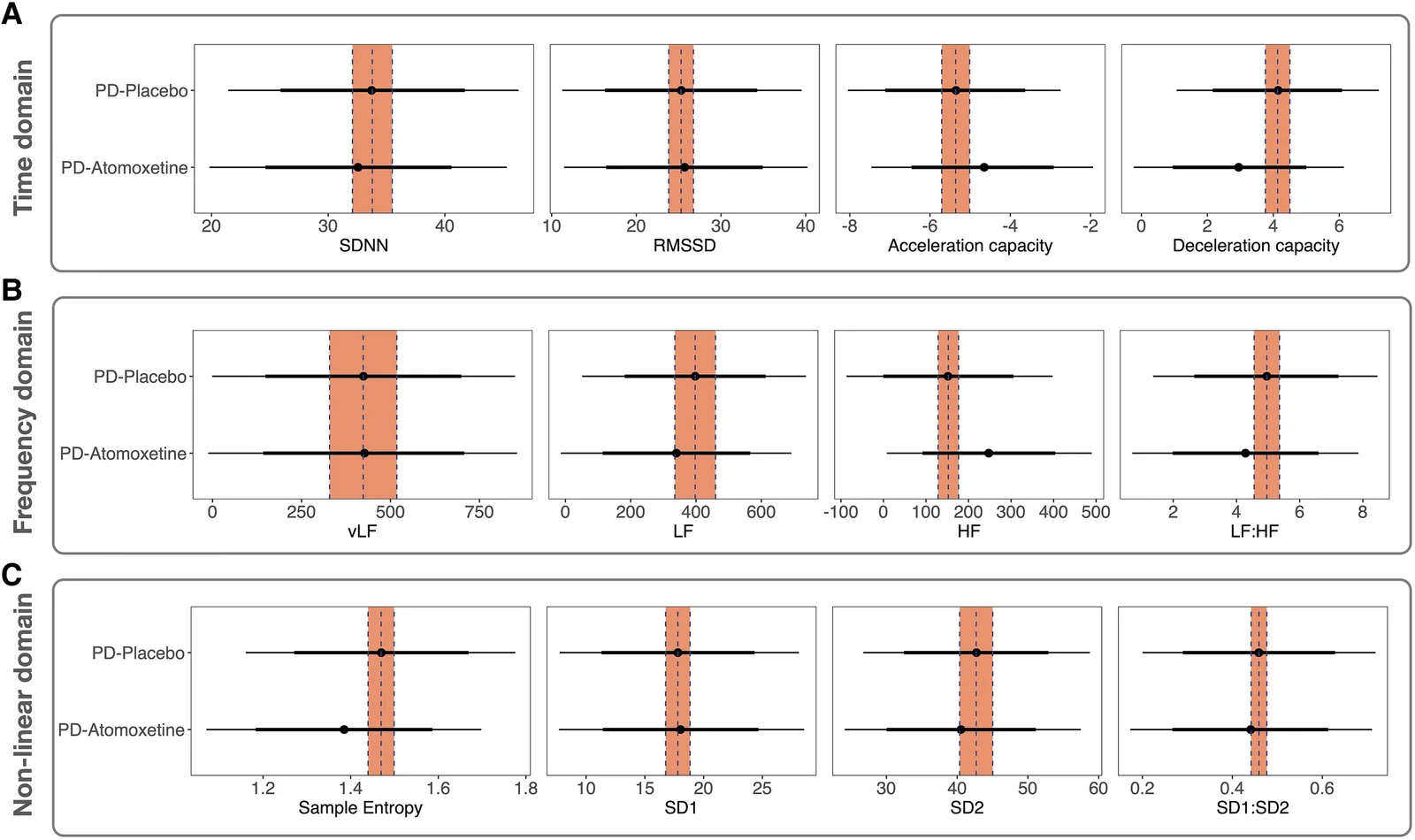
**HRV comparisons on placebo versus atomoxetine in Parkinson’s disease.** Distributions (thin black lines) show the Bayesian posterior estimates of HRV scores for participants with Parkinson’s disease on placebo versus atomoxetine (*N* = 15 participants), for (**A**) Time domain, (**B**) Frequency domain, (**C**) Non-linear domain. Estimates were obtained from Bayesian linear mixed-effects regression models with weakly informative Student’s *t* priors centred on the placebo-group mean for each metric. The dotted vertical line indicates the placebo-group mean; thick horizontal bars show the 75% CI and thin bars the 95% CI. The shaded orange column denotes the ROPE, defined as ±0.1 × σ of the control group for each metric. Standardized effect sizes (β/σ) and 95% CIs for each metric are reported in the Results. SD1 = standard deviation of ellipse width on the Poincaré plot; SD2 = standard deviation of ellipse length on the Poincaré plot; SD1:SD2 = SD1-to-SD2 ratio; PD = Parkinson’s disease.

HRV metrics for the three groups are shown in Fig. 1 (see Supplementary Table 2 for group means). For the Parkinson’s disease atomoxetine versus placebo comparisons, the posterior distributions of HRV estimates and ROPE are shown in Fig. 2. Overall, there was good evidence in favour of the null hypothesis of no difference between the atomoxetine versus placebo sessions.

In the time domain, between the atomoxetine and placebo conditions, there was convincing evidence for no difference in both SD of NN intervals (SDNN) (effect size = −0.12, 95% CI = −8.65 to 6.00) and root mean square of successive differences (RMSSD) (effect size = 0.03, 95% CI = −8.08 to 8.76), with the posterior means falling within the ROPE. Evidence was also in favour of no group difference for both Acceleration (effect size = 0.30, 95% CI = −0.92 to 2.26) and Deceleration (effect size = −0.46, 95% CI = −2.92 to 0.61) capacity, with the CIs of the two groups largely overlapping.

In the frequency domain, there was clear evidence for no difference in very-low-frequency (vLF) power (effect size = 0.005, 95% CI = −249.73 to 254.08) and low-frequency (LF) power (effect size = −0.22, 95% CI = −248.78 to 139.84), with the group means falling within the ROPE. Evidence was also indicative of a lack of group difference for high-frequency (HF) power (effect size = 0.46, 95% CI = −38.02 to 225.46) and the LF:HF ratio (effect size = −0.39, 95% CI −1.94 to 0.59), given the considerable overlap of the CIs.

In the non-linear domain, there was good evidence of no difference for SD1 (effect size = 0.03, 95% CI = −5.69 to 6.21) and SD2 (effect size = −0.16, 95% CI = −11.63 to 7.31) with the means falling within the ROPE. Evidence was also suggestive of a lack of group difference for both sample entropy (effect size = −0.33, 95% CI = −0.25 to 0.09) and SD1:SD2 (effect size = −0.09, 95% CI = −0.17 to 0.13), with largely overlapping CIs.

#### Evidence for altered HRV in Parkinson’s disease compared to healthy controls

**Figure 3:**
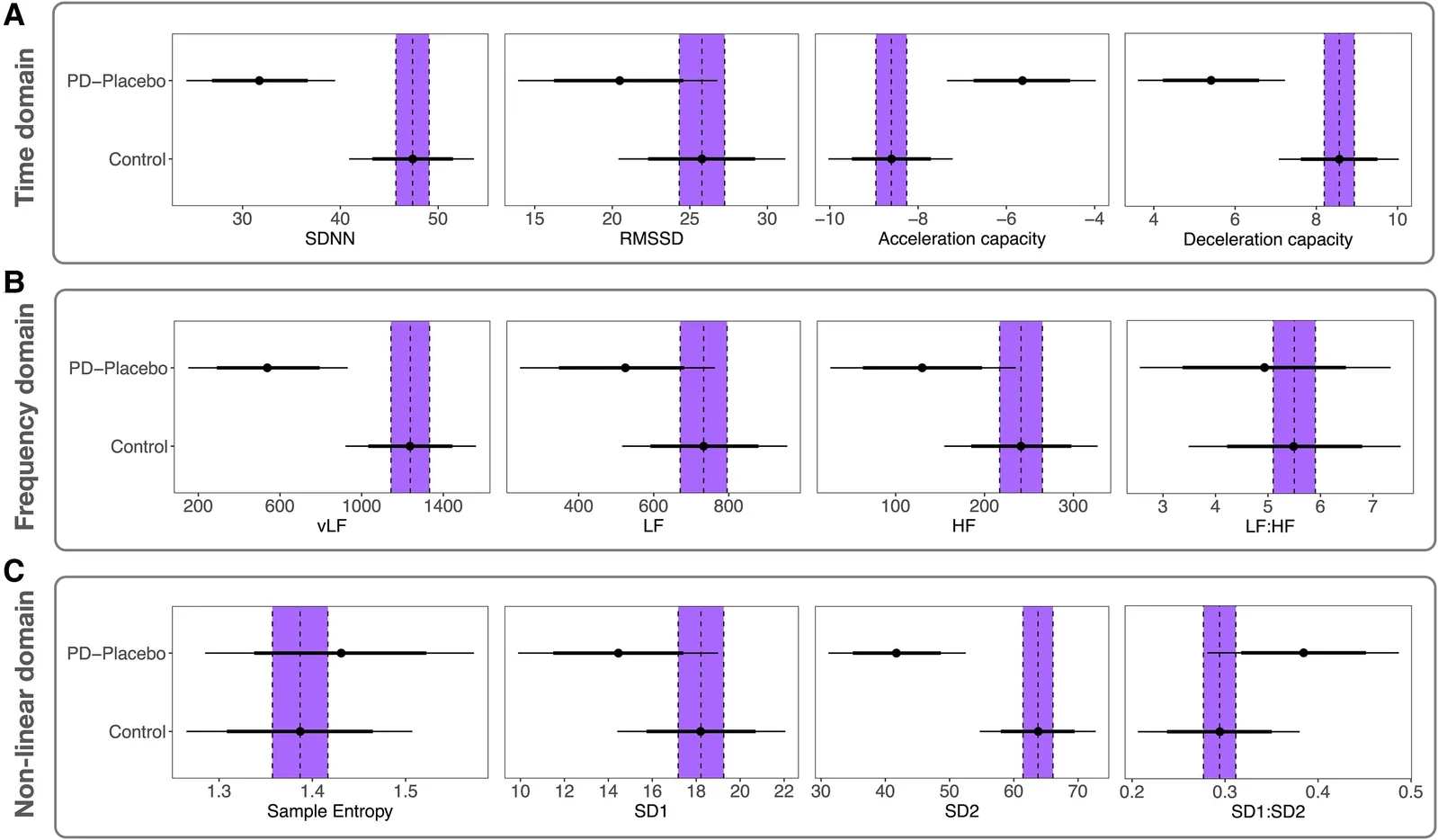
**HRV comparisons between Parkinson’s disease (on placebo) and healthy controls.** Distributions (thin black lines) show the Bayesian posterior estimates of HRV scores for participants with Parkinson’s disease on placebo (*n* = 15) and healthy controls (*N* = 22), for (**A**) Time domain, (**B**) Frequency domain, (**C**) Non-linear domain. Estimates were obtained from Bayesian linear regression models with weakly informative Student’s *t* priors centred on the control-group mean for each metric. The dotted vertical line indicates the control-group mean; thick horizontal bars show the 75% CI and thin bars the 95% CI. The shaded purple column denotes the ROPE, defined as ±0.1 × σ of the control group for each metric. Standardized effect sizes (β/σ) and 95% CIs for each metric are reported in the Results. SD1 = standard deviation of ellipse width on the Poincaré plot; SD2 = standard deviation of ellipse length on the Poincaré plot; SD1:SD2 = SD1-to-SD2 ratio; PD = Parkinson’s disease.

We found evidence of group differences in HRV scores across multiple measures in participants with Parkinson’s disease on placebo compared to healthy controls (Fig. 3).

In the time domain, there was strong evidence of group differences in the Parkinson’s group versus controls for SDNN (effect size = −1.03, 95% CI = −25.29 to −5.75), acceleration capacity (AC: effect size = 0.87, 95% CI = 0.76 to 5.15) and deceleration capacity (DC: effect size = −0.88, 95% CI = −5.46 to −0.79), with the Parkinson’s disease group mean and the 95% CI falling entirely outside of the ROPE and non-overlapping with the control group. In contrast, there was evidence in favour of no group difference for RMSSD (effect size = −0.40, 95% CI = −13.62 to 3.09), given the considerable overlap of the CIs.

In the frequency domain, there was good evidence for reduced vLF power in Parkinson’s disease patients compared to controls (effect size = −0.90, 95% CI = −1194.72 to −190.02), with the mean of the posterior falling well outside the ROPE and non-overlapping CIs. In contrast, evidence was in favour of no group difference for LF power (effect size = −0.38, 95% CI = −580.79 to 47.77), HF power (effect size = −0.53, 95% CI = −247.41 to 22.27) and LF:HF score (effect size = −0.12, 95% CI = −3.62 to 2.48), with CIs falling within the ROPE and overlapping.

In the non-linear domain, we found strong evidence of reduced SD2 in the Parkinson’s disease group (effect size = −1.04, 95% CI = −35.80 to −8.51), with no overlap in the CIs. Whereas evidence suggested a lack of group differences in SD1 (effect size = −0.39, 95% CI = −9.52 to 2.27), SD1:SD2 (effect size = 0.41, 95% CI = −0.04 to 0.22) and sample entropy (effect size = 0.14, 95% CI = −0.14 to 0.23), with overlapping CIs that crossed into the ROPE.

#### Caudal locus coeruleus integrity relates to measures of HRV in Parkinson’s disease

**Figure 4:**
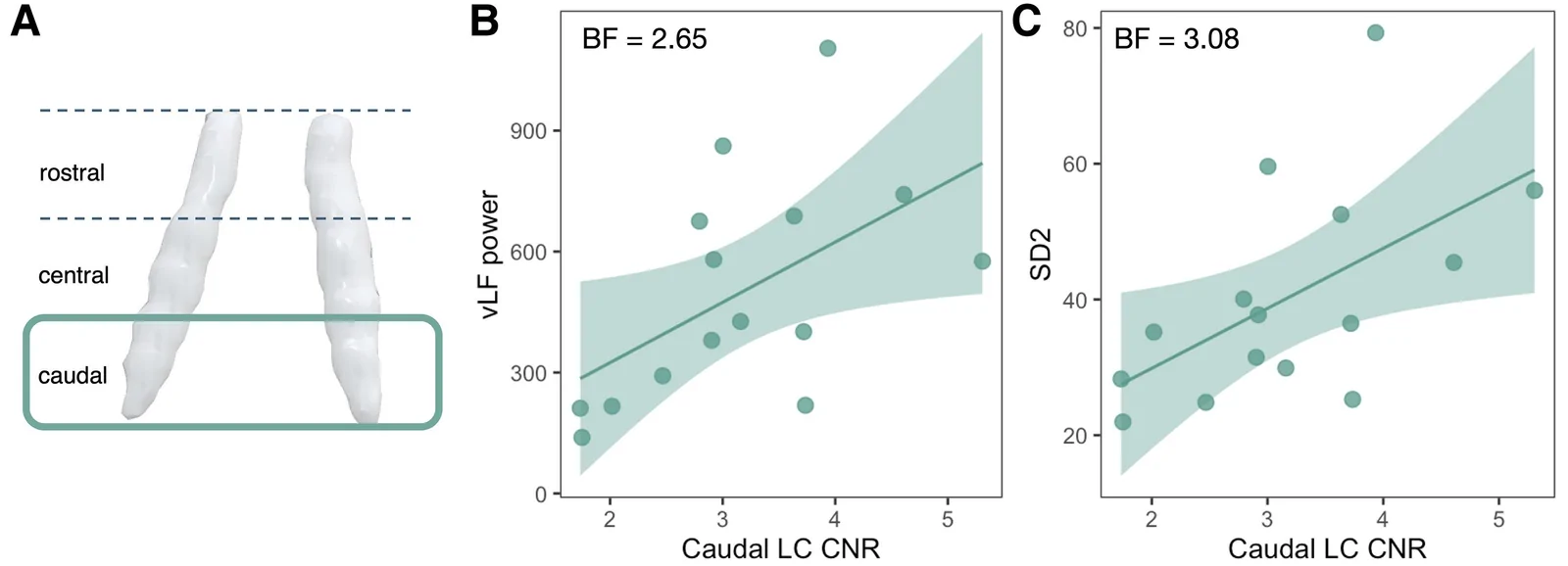
**Relationship between locus coeruleus integrity and HRV.** (**A**) We extracted the CNR from the caudal portion of the locus coeruleus as a measure of integrity in Parkinson’s disease participants. (**B**) Relationship between vLF power and caudal locus coeruleus integrity in Parkinson’s disease, tested using Bayesian linear correlation, median *r* = 0.38, BF = 2.65. (**C**) Relationship between SD2 and caudal locus coeruleus integrity in Parkinson’s disease, tested using Bayesian linear correlation, median *r* = 0.41, BF = 3.08. Data points represent individual participants (*N* = 15 people with Parkinson’s disease). LC = locus coeruleus.

In the exploratory *post hoc* analysis, we tested whether there was a relationship between caudal locus coeruleus integrity and the HRV metrics that were altered in Parkinson’s disease relative to controls (i.e. SDNN, AC, DC, vLF and SD2). We found moderate evidence for a positive correlation between caudal locus coeruleus CNR and both vLF power (*r* median = 0.38, BF = 2.65) and SD2 (*R* median = 0.41, BF = 3.08) (See Fig. 4). That is, higher caudal locus coeruleus integrity was related to stronger power in the vLF band and larger SD2. We did not find evidence of a relationship between caudal locus coeruleus CNR and SDNN (*r* median = 0.34, BF = 1.78), AC (*r* median = −0.34, BF = 1.76) or DC (*r* median = 0.21, BF = 0.822).

## Discussion

We report evidence for the preservation of short-term HRV following 40 mg of atomoxetine in people with Parkinson’s disease. Several measures of HRV were altered in Parkinson’s disease relative to healthy controls. Consistent with the role of the locus coeruleus in modulating cardiac function, reduced caudal locus coeruleus integrity was associated with select indices of abnormal HRV.

Atomoxetine did not alter short-term HRV in people with Parkinson’s disease or change pulse rates and sitting blood pressure. There was a small elevation of diastolic blood pressure lying down. The absence of cardiac effects we report here is important in considering safety of the drug, made especially relevant by the evidence that the same atomoxetine dose (40 mg) is sufficient to modulate behaviour and restore brain activity and connectivity in Parkinson’s disease.^4-6,8,49^ Encouragingly, this suggests that 40 mg of atomoxetine may benefit cognitive and psychiatric symptoms in Parkinson’s disease without affecting cardiac and cardiovascular regulation. The noradrenergic system has a diverse influence on cognitive processes and behavioural regulation.^50-52^ Disruptions to this system contribute to cognitive and psychiatric symptoms in neurodegenerative diseases of ageing.^2^ The pro-cognitive effects of atomoxetine are mediated by its actions on post-synaptic α2A receptors in the prefrontal cortex.^53^ With growing recognition of a noradrenergic subtype in Parkinson’s disease,^1^ there is scope for repurposed noradrenergic drugs, such as atomoxetine, to form part of a personalized treatment approach in Parkinson’s disease and related conditions.^3^

It is important to note that our atomoxetine intervention was not hypothesized to restore HRV. Global cardiovascular effects of atomoxetine are mediated by opposing peripheral and central effects. Peripherally, atomoxetine increases noradrenaline concentration at peripheral sympathetic synapses, which can lead to increased blood pressure^54^ (a well-documented side-effect^13^). Centrally, atomoxetine can elevate brainstem noradrenaline and act on α2 receptors to suppress sympathetic outflow.^55^ Taken together, there is no strong theoretical grounding to suspect that atomoxetine would have restorative effects on cardiovascular function. Moreover, these cardiovascular effects invite further caution when using the drug in a population with extensive central and peripheral autonomic dysfunction. In this context, our null finding is particularly relevant, as we saw no detectable perturbation of HRV at the 40 mg dose used.

Previous work has identified HRV abnormalities in Parkinson’s disease compared to healthy controls, although there is considerable variability in findings across and within studies.^24,56^ Heart rate is regulated by the sympathetic and parasympathetic autonomic nervous systems, and their interaction with cardiovascular (e.g. baroreceptors) and respiratory regulatory mechanisms.^57^ Optimal levels of HRV mean an organism can adaptively respond to whatever their current context requires, with reduced HRV a feature of many health problems and diseases.^58^ We found reduced HRV in Parkinson’s disease across measures from the time and frequency domains (i.e. SDNN and vLF) and in a complexity measure (SD2). Measures of deceleration-related and acceleration-related capacities of heart rate were also altered compared to controls. Reductions in various HRV measures, as well as a reduced DC, have been previously shown in Parkinson’s disease,^24,59-61^ consistent with the widespread pathological changes that impact both central and peripheral regulators of HRV.^19^ Reduced HRV may be a prodromal feature of Parkinson’s disease^62^; greater reductions in HRV have been associated with disease severity^60,63^ and with specific symptoms, including mild cognitive impairment,^64^ anxiety^65^ and freezing of gait.^66^ Our finding that atomoxetine, when administered to a population whose autonomic system is already compromised, does not further perturb HRV is therefore directly relevant to its clinical safety profile in Parkinson’s disease.

The locus coeruleus plays a key role in cardiac regulation. It provides direct noradrenergic modulation over preganglionic sympathetic neurons in the intermediolateral cell column of the spinal cord and over preganglionic parasympathetic vagal neurons, including the dorsal motor nucleus of the vagus and the nucleus ambiguus.^22^ The locus coeruleus also exerts influence via projections to sympathetic premotor nuclei, including those in the rostroventrolateral medulla that contain an intrinsic pacemaker activity and are involved in maintaining blood pressure and heart rate.^67^ Focusing on the measures that were altered in people with Parkinson’s disease compared to controls, we found an association between caudal locus coeruleus CNR and certain HRV measures (i.e. vLF and complexity). Specifically, reduced caudal locus coeruleus integrity was associated with reduced HRV in those measures. Locus coeruleus contrast on neuromelanin-sensitive imaging has also been associated with HF HRV in healthy people.^68^ Taken together, neuromelanin-sensitive imaging of the locus coeruleus is emerging as a useful biomarker in psychiatry and neurology that can signal both cognitive and autonomic changes,^69^ which may help predict the patients who will respond best to noradrenergic therapy.^7^

Our key finding is that short-term HRV was preserved in people with Parkinson’s disease after an atomoxetine challenge. This extends previous work, which had demonstrated that atomoxetine was not associated with significant alterations in blood pressure and pulse rate. Here, we demonstrate stability under atomoxetine on a comprehensive set of HRV measures that capture the dynamic regulation of autonomic state. This favourable signal will benefit from replication in a larger sample with longer-duration ECG recordings, and chronic atomoxetine administration. Another future avenue would be to determine whether background anti-parkinsonian dopaminergic medication modulates the effect of atomoxetine on HRV, which could be tested in a larger sample with sufficient variation in dosing regimen. Nevertheless, the results are timely given the growing interest in repurposing atomoxetine to treat cognitive and psychiatric symptoms in neurodegenerative diseases of ageing—as demonstrated by the work in Parkinson’s disease, current trials in Parkinson-plus disorders^70^ and mild cognitive impairment,^10^ and recent calls for trials in Alzheimer’s disease.^71,72^ Our findings are in favour of a safe cardiovascular profile for atomoxetine in Parkinson’s disease, lending further support to noradrenergic modulation as a viable treatment strategy for neuropsychiatric symptoms in Parkinson’s disease and related disorders.

## Supplementary Material

fcag338_Supplementary_Data

## Data Availability

Code and data to reproduce manuscript figures and statistical analyses are openly available https://osf.io/q2xtm.
